# TNFα is responsible for the canonical offspring number-size trade-off

**DOI:** 10.1038/s41598-019-38844-9

**Published:** 2019-03-14

**Authors:** S. O. Maslennikova, L. A. Gerlinskaya, G. V. Kontsevaya, M. V. Anisimova, S. A. Nedospasov, N. A. Feofanova, M. P. Moshkin, Y. M. Moshkin

**Affiliations:** 1 Federal Research Center Institute of Cytology and Genetics, Siberian Branch of the Russian Academy of Sciences, Novosibirsk, Russia; 2 Lomonosov Moscow State University, Moscow, Russia; 3 Engelhardt Institute of Molecular Biology, Russian Academy of Sciences, Moscow, Russia; 4 Research Institute of Fundamental and Clinical Immunology, Novosibirsk, Russia; 5 Institute of Molecular and Cellular Biology, Siberian Branch of the Russian Academy of Sciences, Novosibirsk, Russia

## Abstract

There is a canonical life-history trade-off between quantity and quality of offspring, but molecular determinants for this are unknown. Here, we show that knockout of tumor necrosis factor (TNF-KO) in mice switched a relation between the number and size of developing embryos from expectedly negative to unexpectedly positive. Depletion of TNFα imbalanced humoral and trophic maintenance of embryo growth during gestation with respect to the litter size. The levels of embryotrophic GM-CSF cytokine and placental efficiency attained positive correlations with the number and size of embryos in TNF-KO females. Thus, TNFα oversees mother’s resource allocations to balance embryo growth with the number of offspring. Consequently, this suggests an intricate link between the number-size trade-off and immunity given a pivotal role of TNFα in immune homeostasis.

## Introduction

A negative relationship between the number and size of offspring is one of the universal life-history trade-offs known for over a century^1–3^. Following Smith-Fretwell (SF) model, investment per offspring (*I*) is proportional to mother’s reproductive resources (*R*), which are partitioned between offspring in a litter (*L*)^4^. Assuming that *I* is converted to embryo growth/mass and if *R* is constant or independent of either *L* or *I*, then the size of offspring is expected to relate negatively to the litter size. The SF relation *I* = *R/L* also suffices for human and other species with single offspring by representing *L* and *R* as fertility rate and reproductive effort per unit time respectively^5^. Through different species *R* scales with adult size following power law as *R* = *a·M*_*female*_^*b*^, where *M*_*female*_ is the mass of a mother, *a* – the normalization constant and *b* – the exponent ranging from ~¾ to ~1 for homeotherms^6,7^. In part, the origin of such allometric scaling could be attributed to the Kleiber’s scaling of a basal metabolic rate (BMR) to the ¾ power of animal’s mass^8–10^. However, in mice the correlations of BMR with litter size or litter weight are negligibly small^11^, and mechanistically it remains unclear what determines the scaling of maternal reproductive effort.

From a perspective of life-history theory, reproductive effort ought to be balanced with self-maintenance costs to maximize the fitness of offspring while minimizing losses in the maternal one. Self-maintenance costs, among others, include expenses for immunity. However, although activation of immune response is a demanding process, immune maintenance is thought to be cheap and a trade-off between immune homeostasis and reproductive effort is a matter of debate^12–15^. To address a role of immune maintenance in reproduction, we surveyed reproductive phenotypes in mice knockout for tumor necrosis factor (TNF-KO) free of specific pathogens. TNFα exerts immense “regulatory authority” on the mammalian immunity. It binds to tumor necrosis factor receptors TNFR1, TNFR2 and, depending on their ratio, triggers either pro- or anti-survival cellular programs through the activation of NF-κB, Death-inducing signaling complex and other signaling cascades^16–19^. To our surprise, knockout of TNFα switched a relation between litter size and embryo weight from a negative one to a positive. This was followed by attaining of positive correlations for the litter size with embryo to placenta weight ratio and the levels of embryotrophic GM-CSF cytokine in amniotic fluid. Thus, TNFα is responsible for restricting of embryo growth with respect to embryo number suggesting a mechanism responsible for the number-size trade-off, which involves a balance between embryotoxic and embryotrophic immune cytokines.

## Results

### TNF-KO affects mother’s resource allocations to offspring and the number-size trade-off

Considering a gaining popularity of pharmacological inhibition of TNFα for treatment of autoimmune disorders including in pregnant women^20–23^, we evaluated reproduction in mice knockout for TNFα (TNF^−/−^)^24^. In the strain used in this study transcriptionally active *neo* cassette has been removed^24–26^. TNF-KO was transferred to C57BL/6 J genetic background and C57BL/6 J was used as a control (TNF^+/+^). Consistent with its function in immunity, knockout of TNFα reduced weights of immune organs: spleen in males and females and thymus in males (see Supplementary Fig. S1). To account for maternal and paternal effects of TNFα on reproduction, control and TNF-KO females were mated with either TNF^+/+^ or TNF^−/−^ males.

**Figure 1:**
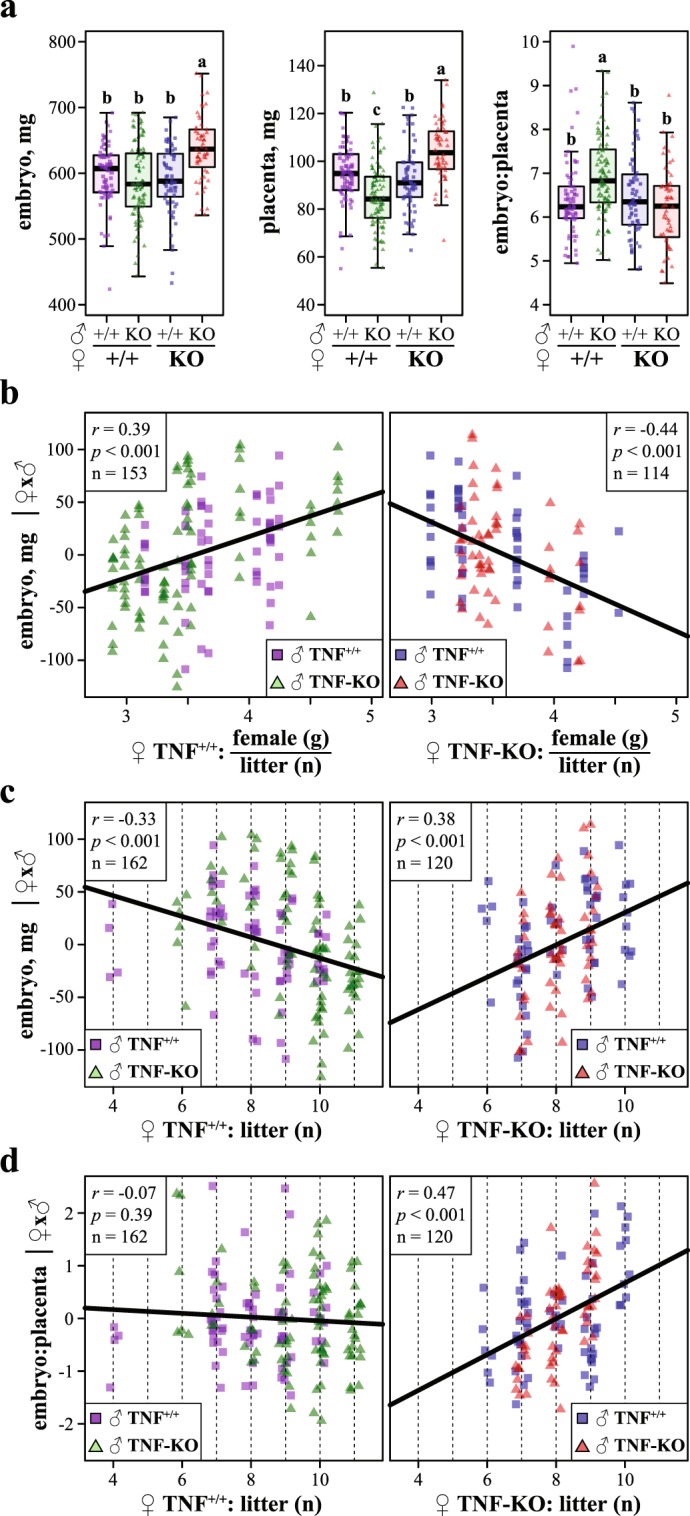
Knockout of maternal TNFα violates the offspring number-size trade-off. (**a**) One-way ANOVA followed by Least Significant Difference test (LSD) showed that embryo and placental weights, and *E*:*P* ratio at stage E16.5 of embryo development were affected by the parental genotype (TNF^+/+^ or TNF-KO). Letters indicate statistically significant differences (p < 0.05) of LSD. Individual and boxplot-summarized values are shown. Boxes correspond to quartile Q1 to Q3 range, lines – medians, and whickers extend to 1.5 interquartile range. Partial correlations of residual embryo weights corrected to the parental genotypes with (**b**) mother weight to litter size ratio and (**c**) the number of offspring for TNF^+/+^ (left panel) and TNF-KO (right panel) dams. Squares and triangles indicate TNF^+/+^ and TNF^−/−^ paternal genotypes respectively and regression lines are shown. For correlations with unadjusted values see Supplementary Fig. S2 and for statistical summary see Supplementary Table S2. (**d**) Correlations of residual embryo to placenta weight ratios (*E*:*P*) corrected to the parental genotypes with the number of offspring. For further details see Supplementary Table S2 and Fig. S2.

In TNF-KO females, the ovulation rates were significantly reduced by ~20% (Table 1, see Supplementary Table S1). Knockout of maternal and/or paternal TNFα increased pre-implantation and total (pre- and post-implantation) embryo losses by ~10% (Table 1, see Supplementary Fig. S1). Despite increased embryo losses for TNF-KO parents, effectiveness of successful gestations (litter size and litter weight at stage E16.5 of embryo development) was higher in matings of TNF^−/−^ males with either TNF^+/+^ or TNF^−/−^ females (Table 1, see Supplementary Table S1). To that, knockout of TNFα in both parents increased embryo weights (Fig. 1a). Increase in the number of live embryos in matings with TNF-KO fathers was attributed to implantation failure in females with low ovulation rates. Maternal genotype had no effect on neither litter size nor litter weight. Together, these results substantiate an intricate role of TNFα in reproduction^27^. Maternal TNFα increases the ovulation rates, while zygotic – decreases the rates of pre-implantation losses, and paternal TNFα facilitates embryo implantation in females with low ovulation rates.

**Table 1:** The effect of maternal and paternal TNFα deficiency on fertility, embryo implantation and survival, and reproductive output (litter size, weight in successful pregnancies) in mice (values are means ± SEM, significant effects are indicated in boldface, see Supplementary Table S1 and Fig. S1 for statistical details).

Parameter:	Parental genotype:			
	♀ TNFα +/+		♀ TNFα ^−^/^−^	
	♂ TNFα +/+	♂ TNFα ^−^/^−^	♂ TNFα +/+	♂ TNFα ^−^/^−^
	**all covered females**			
N of covered females	12	14	13	14
• ovulated eggs (n)	**8.65 ± 0.5**		**6.7 ± 0.57**	
• implanted embryos (n)	6.58 ± 1.12	7.14 ± 1.29	5.61 ± 1.25	4.29 ± 1.2
• live embryos (n)	5.92 ± 1.02	6.5 ± 1.2	4.92 ± 1.12	4.00 ± 1.12
• pre-implantation losses (%)	14.13 ± 3.63	**24.81 ± 3.74**	**24.74 ± 4.38**	**28.57 ± 4.93**
• post-implantation losses (%)	10.13 ± 3.4	9.0 ± 2,86	12.33 ± 3.85	6.7 ± 3.22
• total losses (%)	22.83 ± 4.38	**31.58 ± 4.03**	**34.02 ± 5.14**	**33.33 ± 5.14**
	**successful pregnancies**			
N of successful pregnancies	10	10	9	7
• litter size (n)	7.1 ± 0.77	**9.1 ± 0.53**	7.11 ± 0.87	**8.0 ± 0.31**
• litter weight (g)	4.24 ± 0.44	**5.35 ± 0.23**	4.2 ± 0.55	**5.1 ± 0.27**

In agreement with mass-scaling of reproductive resources, litter weights at stage E16.5 were highly correlated with mothers’ weights for TNF^+/+^ (*r* = 0.71, *p* < 0.001, n = 20) and TNF^−/−^ (*r* = 0.73, *p* < 0.05, n = 16) females. Likewise, embryo weights are expected to relate positively to maternal investments per offspring expressed as a ratio of mother’s weight to the litter size^5^. Although this was a case for offspring of control females, this relation changed to a negative for offspring of females deficient for TNFα (Fig. 1b, see Supplementary Fig. S2 and Table S2). As a result, in contradiction to the number-size trade-off, embryo weights increased with increasing litter size in TNF-KO dams (Fig. 1c, see Supplementary Fig. S2 and Table S2). To that, embryo to placenta weight ratio (*E*:*P*) correlated positively with the number of offspring in TNF-KO females, but not in the control females (Fig. 1d, see Supplementary Fig. S2 and Table S2). *E*:*P* serves as a proxy to placental efficiency and higher *E*:*P* associates with increased nutrient transfer from mother to embryo^28^. Thus, we conclude that knockout of TNFα leads to imbalanced mother’s resource allocations to offspring with respect to their abundance during gestation, thereby overriding the number-size trade-off.

### TNFα balances GM-CSF levels in response to variations in the number and size of offspring

Optimal embryo growth is achieved through a balance between embryotrophic and embryotoxic cytokines^29^. TNFα falls more into the latter group and, therefore, we wondered if its depletion would affect the levels of embryotrophic cytokines. Granulocyte-macrophage colony stimulating factor (GM-CSF) is one of such cytokines. It is thought to be a master paracrine regulator of embryo health, which promotes blastocyst implantation, mother to embryo nutrient transfer, and, as a result, embryo growth and survival^29,30^. Although GM-CSF is virtually absent from the systemic circulation, it is secreted by epithelium of reproductive tract allowing its detection in amniotic fluid^29–31^.

**Figure 2:**
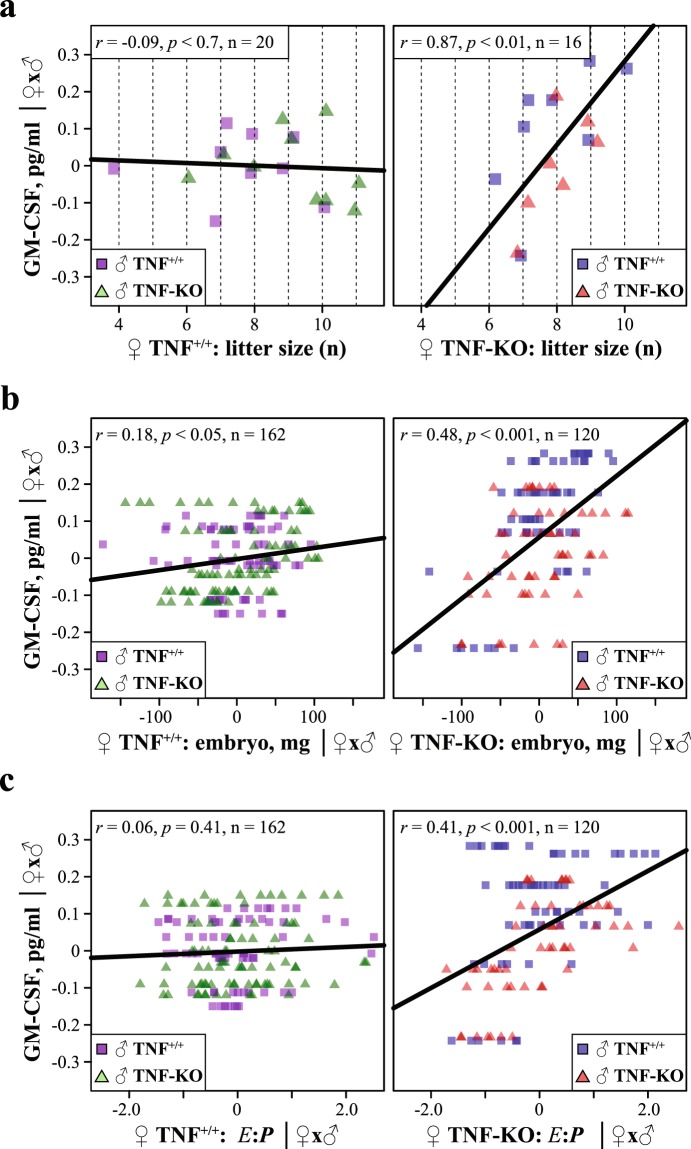
TNFα limits the levels of amniotic GM-CSF in response to increasing number of embryos. Partial correlations of residual GM-CSF concentrations corrected to the parental genotypes for TNF^+/+^ (left panel) and TNF-KO (right panel) females with (**a**) litter size, (**b**) embryo weights adjusted to the parental genotypes and (**c**) adjusted *E*:*P* weight ratios. For correlations with unadjusted values see Supplementary Fig. S3 and for statistical summary see Supplementary Table S3.

Overall, knockout of TNFα had little effect on concentrations of GM-CSF assayed by ELISA on day 16.5 of gestation in amniotic fluid (see Supplementary Fig. S3). However, GM-CSF levels were significantly increased in TNF-KO females mated with TNF-KO males, which paralleled an increase in embryo weights specific for this mating group (Figs 1a, see Supplementary Fig. S3). In the control females, GM-CSF was uncorrelated with the number of offspring, but in TNF-KO dams GM-CSF levels raised significantly with increasing litter size (Fig. 2a, see Supplementary Fig. S3 and Table S3). Likewise, GM-CSF attained positive correlations with embryo weight and placental efficiency (*E*:*P*) in females deficient for TNFα (Fig. 2b,c, see Supplementary Fig. S3 and Table S3). This suggests that maternal TNFα restricts GM-CSF secretion to limit embryo growth in large litters, thus, balancing mother’s reproductive effort with respect to the number of offspring. Knockout of maternal TNFα alleviates this restriction turning the number-size trade-off on a dime.

### Hormonal maintenance of gestation is altered in the absence of TNFα

Proper gestation also requires a balance in hormonal milieu^32^. Thus, along with GM-CSF cytokine, we assessed progesterone, corticosterone and testosterone concentrations in serum and amniotic fluid. On average, the levels of amniotic hormones and blood plasma progesterone were unaffected by the knockout of TNFα, while circulating corticosterone and testosterone changed depending on the parental genotype (see Supplementary Fig. S4). Concentration of blood plasma testosterone was lower in TNF-KO females as compared to the control. Notably, however, the levels of serum corticosterone levels were increased in TNF-KO females mated with TNF-KO males suggesting mobilization of mother’s resources to support gains in embryo weights (Fig. 1a, see Supplementary Fig. S4). Although exposure to glucocorticoids modulates mother to fetus nutrient transfer in various ways^33^, a possible growth limiting effect of corticosterone might be compensated for by an increase in GM-CSF cytokine (see Supplementary Fig. S3).

**Figure 3:**
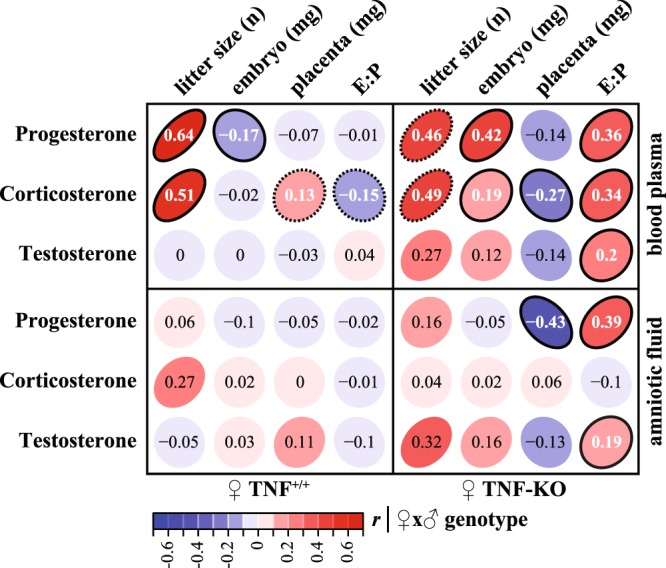
TNFα affects hormonal maintenance of gestation. Correlation heatmap of partial correlations of parental genotype adjusted hormone (progesterone, corticosterone, testosterone) concentrations in serum (top panel) and amniotic fluid (bottom panel) with litter size, embryo and placental weights and *E*:*P* corrected for parental genotypes for TNF^+/+^ (left panel) and TNF-KO (right panel) females. For summarized statistics of hormone levels and correlations with unadjusted values see Supplementary Fig. S4 and Tables S4, S5. Significant correlations (p < 0.05) are outlined. Dashed lines indicate marginally significant correlations (p < 0.1).

Importantly, knockout of maternal TNFα altered a pattern of correlations between the hormones and the number of offspring, embryo weight, placental weight and *E*:*P* ratio (Fig. 3, see Supplementary Fig. S4, Tables S4, S5). First, we noted that all measured hormones, except for corticosterone in amniotic fluid, related positively to *E*:*P* ratio in TNF-KO females. To that, correlations of placental weight with serum corticosterone and amniotic progesterone were also altered. Next, serum progesterone and corticosterone levels attained positive correlations with embryo weights in TNF-KO females mirroring the inversion of the number-size trade-off. These suggest that with respect to the number of offspring TNFα modulates embryotrophic potential of progesterone, energy recruitment by corticosterone as well as hormonal control of placental mother-to-fetus energy flux. Depletion of TNFα, in turn, might imbalance hormone-mediated mobilization and allocation of maternal resources to fetus, thereby alleviating litter size-dependent embryo growth restriction.

### TNFα also affects male’s reproductive function

Paternal effects of TNFα on embryo implantation (Table 1) could either be attributed to seminal transfer of TNFα or, non-mutually exclusive, to alterations in male’s reproductive system and sperm quality^34^. Indeed, we noted an increase in testosterone levels, though with large variations, in males deficient for TNFα (see Supplementary Fig. S5). The weights of androgen-dependent tissues: testis, seminal vesicle - were unaffected, while caudal epididymis was enlarged in TNF-KO males (see Supplementary Fig. S5). Percentages of spermatogonia, spermatocytes and spermatids were comparable between the control and TNF-KO, but the number of spermatozoa per mass of caudal epididymis were lower in TNF-KO males. Percentage of motile spermatozoa was also decreased in males deficient for TNFα (see Supplementary Fig. S5). On contrary, however, curvilinear and straight-linear velocities were higher for spermatozoa of TNF-KO males (see Supplementary Fig. S5). Spermatozoon dynamics depends on its head shape and size. Although, head elongation, a ratio of minor to major axis was unaffected, head area was lower for spermatozoa of TNF-KO males (see Supplementary Fig. S5). From this, we conclude that knockout of TNFα affects sperm quality, which, as a result, might have an impact on embryo quality and implantation success^34^.

## Discussion

In conclusion, our results revealed a novel function for pro-inflammatory cytokine TNFα in the trade-off between the number and size of offspring. Following Smith-Fretwell reasoning^4^, the violation of this trade-off in mouse dams deficient for TNFα immediately implies imbalanced reproductive effort and mother-to-fetus resource allocations (Fig. 1b,c). Consequently, taking into account a role of TNFα in maintenance of immune homeostasis^16–19^, this suggests an intimate link between the two canonical life history trade-offs, namely the number-size and reproduction-immunity trade-offs. Maintenance of immunity is thought to be cheap^12–14^. However, given that mice in our experiments were housed in the specific pathogen free environment, this indicates that reproduction-immunity trade-off also holds in the absence of immune activation.

In previous experiments, we noted a violation of the number-size trade-off in females mated with immune-primed males, but there was no mechanistic explanation to this^35^. Here, we propose that the depletion of TNFα alleviates litter size dependent restrictions on the levels of embryotrophic cytokine GM-CSF (Fig. 2a). This, in turn, might increase placental efficiency and promote embryo growth in females pregnant with large litters^29,30^. Thus, a balance between embryotoxic TNFα and embryotrophic GM-CSF cytokines is likely to be involved in a control of the number-size relation. In addition, it has to be noted that, similarly to TNFα, GM-CSF is required for immune homeostasis^31^, providing further evidence for a cross talk between reproduction-immunity and the number-size trade-offs.

The lack of maternal TNFα leads to imbalanced hormonal milieu during gestation in relation to embryo growth and placental efficiency (Fig. 3). Steroid hormones exert pleotropic effects on mother’s resource mobilization, mother-to-fetus nutrient transfer and, as a result, embryo growth and maternal adaptation to pregnancy^32,36–38^. In turn, such imbalance caused by the absence of TNFα might alter the expectedly negative relations between embryo growth and the number of embryos in a litter. To that, progesterone, corticosterone and testosterone are all implicated in modulation of immunity during gestation^39^. Again, this indicates that immune homeostasis maintained by cytokines and hormones might balance the reproductive effort with respect to the number of offspring.

TNFα and its receptors TNFR1/TNFR2 are members of an ancient tumor necrosis factor superfamily TNFSF/TNFRSF maintaining remarkable functional conservation in animals for 550 My^40^. This implies that TNFSF/TNFRSF could be broadly involved in the number-size trade-off across species. The number-size trade-off also applies to monotocous animals including human^5^. Whether in human TNFα is implicated in maternal resource allocations to offspring in accordance with the fertility rate remains to be seen, it is important to note that anti-TNFα therapies of autoimmune disorders already prescribed to pregnant women^20–23^. Thus, our results call for careful evaluation of potential risks of such therapies to mothers and infants.

## Methods

### Mice and experimental mating groups

Mice knockout for TNFα (TNF^−/−^) transferred to C57BL/6 J genetic background were described previously^24^. C57BL/6 J strain (000664, the Jackson Laboratory) was used as a control (TNF^+/+^). Animals free of species-specific pathogen (SPF) were housed in individually ventilated cages OptiMICE (Animal Care Systems Inc., USA) at 22–24 °C, 40–50% humidity with artificial photoperiod (14 L:10 D). Animals had *ad libitum* access to water and granulated complete feed for mice (SSNIFF, Germany). For matings of animals aged 8–10 weeks, two virgin females (TNF^+/+^ or TNF^−/−^) were placed after turning light off with a male (TNF^+/+^ or TNF^−/−^), which comprised 4 mating groups. Females were examined for a vaginal plug daily during the next 5 days. When the vaginal plug was detected, this was considered as day 0 of pregnancy.

### Reproductive phenotyping of gestation

Females were decapitated on day 16.5 of pregnancy, embryos were removed from uterus, placenta and fetal membranes were removed. Embryos and their placentas were weighed along with females and their immune organs: thymus and spleen. To estimate ovulation, and pre- and post-implantation embryo losses, the numbers of corpora lutea (CL), live embryos (LE) and resorptive embryos (RE) were counted (Table 1, see Supplementary Fig. S1).

To estimate humoral maintenance of gestation blood and amniotic fluid samples were collected from each female. Samples of amniotic fluid were pulled from each fetus for each female. Hormone concentrations in blood plasma and amniotic fluid were determined by enzyme-linked immunosorbent assay (ELISA) using “Testosteron-IFA” and “Progesterone-IFA” kits (“HEMA” Ltd., Russia), and “Corticosterone ELISA Kit” (Enzo Life Sciences Inc., USA). Measurements were carried out without preliminary extraction according to manufacturer’s instructions and as previously described^35^. The minimal testosterone concentration, which could be reliably detected, was 0.087 ng/ml, and the intra- and inter-assay coefficients of variation were 8.2% and 5.6% respectively. The minimal detectable progesterone concentration was 0.15 ng/ml, and the intra- and inter-assay coefficients of variation were 7.6% and 4.3% respectively. For corticosterone, the minimal concentration was 0.027 ng/ml and the intra- and inter-assay coefficients of variation were 8.4% and 8.2% respectively.

GM-CSF concentrations were measured in amniotic fluid by mouse GM-CSF ELISA set (BD Biosciences, USA). Measurements were performed in accordance with manufacturer’s instructions using vertical scanning plate spectrophotometer iMark^TM^ (Bio-Rad, USA). The intra- and inter-assay coefficients of variation were 5.2% and 3.9% respectively. Blood GM-CSF levels were below detection limit.

### Reproductive phenotyping of males

Males after 5 days of joint maintenance with females were phenotyped. Prior to euthanasia, blood samples were collected from the retroorbital sinus and the levels of testosterone were assayed by ELISA. After euthanasia (craniocervical dislocation), body weight, weights of testes, epididymis, seminal vesicles, spleen and thymus were determined. Body weights were comparable for TNF^+/+^ and TNF^−/−^ males allowing for direct comparisons of androgen-dependent and immune organs.

The caudal epididymis sections were homogenized in 500 μl of Hank’s Basic Salt Solution without calcium and magnesium (Sigma-Aldrich, USA) and incubated at 37 °C for 20 minutes. Suspension was filtered through a capron filter and gently shaken. Concentration, mobility and morphology of spermatozoa were assessed with automatic semen analyzer MouseTraxx (Hamilton Thorne, USA) according to manufacturer’s protocol.

Testes were fixed in 10% neutral formalin. Fixed samples were dehydrated in a series of ethanol solutions of ascending concentrations and cleared with xylene. Paraffin blocks were made using HistoStar (Thermo Fisher Scientific, USA). Rotary semiautomatic microtome Microm HM 340E (Thermo Fisher Scientific, USA) was used to prepare 3 μm sections. The sections were deparaffinated and stained with Ehrlich’s hematoxylin and eosin. Spermatogonia, spermatocytes and first generation of spermatids were counted in 15 random transverse sections of seminiferous tubules.

### Statistics

All data were processed and analysed with R (http://cran.r-project.org) using the standard library along with Companion to Applied Regression package (car; http://cran.r-project.org/web/packages/car/index.html) for type II ANOVA. Models were specified through lm function of R according to the manual. Partial correlations (*r* | ♀ × ♂ genotype) were calculated by taking the residuals of variables of interest regressed to the parental genotype (maternal and paternal: TNF^+/+^ or TNF^−/−^).

### Ethic statement

All experiments were conducted at the Centre for Genetic Resources of Laboratory Animals at the Institute of Cytology and Genetics, SB RAS (RFMEFI61914X0005 and RFMEFI61914X0010). All experiments were performed in accordance with protocols and guidelines approved by the Animal Care and Use Committee Federal Research Centre of the Institute of Cytology and Genetics, SB RAS operating under standards set by regulations documents Federal Health Ministry (2010/708n/RF), NRC and FELASA recommendations. Experimental protocols were approved by the Bioethics Review Committee of the Institute of Cytology and Genetics.

## Supplementary information

Supplementary Information

## Data Availability

All data generated and analysed during this study are included in this published article and its Supplementary Information.
