# Supplementary material for: TNFα is responsible for the canonical offspring number-size trade-off
Source: Sci Rep. 2019 Mar 14;9:4568. doi: 10.1038/s41598-019-38844-9 (PMC6418207; doi:10.1038/s41598-019-38844-9)
Supplement: Supplementary file 1 — Supplementary Information [file 41598_2019_38844_MOESM1_ESM.pdf]

Supplementary Information to

TNFα is responsible for the canonical offspring number-size trade-off

S.O. Maslennikova[1], L.A. Gerlinskaya[1], G.V. Kontsevaya[1], M.V. Anisimova[1], S.A. Nedospasov[2,3], N.A. Feofanova[4], M.P. Moshkin[1,*], Y.M. Moshkin[1,5,*]

[1]Federal Research Center Institute of Cytology and Genetics, Siberian Branch of the Russian Academy of Sciences, Novosibirsk, Russia

[2]Lomonosov Moscow State University, Moscow, Russia

[3]Engelhardt Institute of Molecular Biology, Russian Academy of Sciences, Moscow, Russia

[4]Research Institute of Fundamental and Clinical Immunology, Novosibirsk, Russia

[5]Institute of Molecular and Cellular Biology, Siberian Branch of the Russian Academy of Sciences, Novosibirsk, Russia

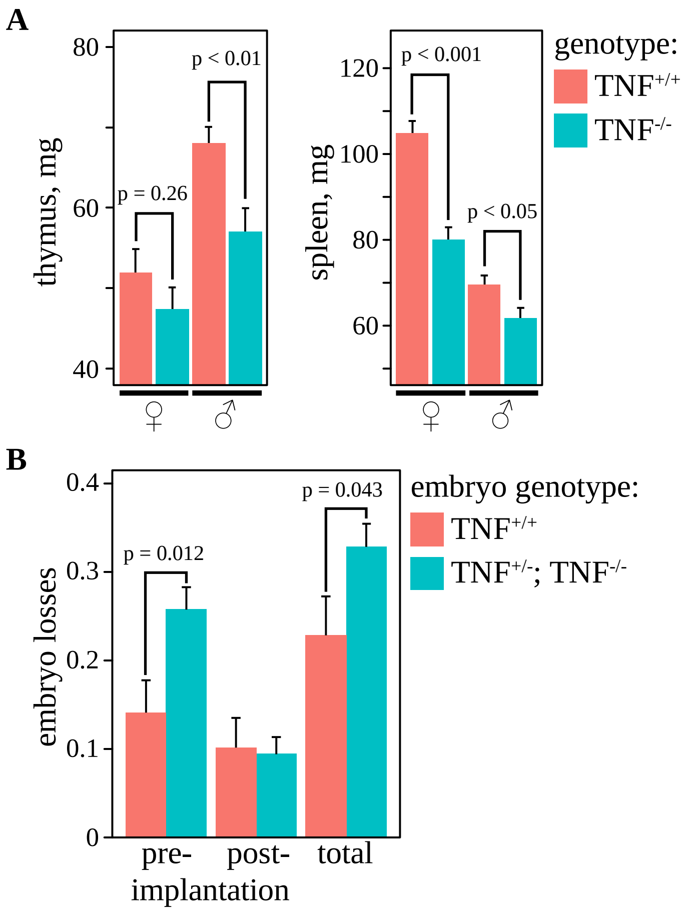

Fig. S1. Zygotic TNFα is required for blastocyst implantation.

A) TNFα functions in the development of immune system. Spleen weights were reduced in both TNF-KO males and females, while thymus weights were lower in TNF-KO males, but not in females. Comparisons were based on Student's t-test.

B) Pre-implantation losses were estimated from the number of ovulated eggs (corpora lutea - CL), and the numbers of live (LE) and resorbed (RE) embryos as 1-(LE+RE)/CL; post-implantation – as RE/(LE+RE), and total – as 1-LE/CL. Embryo losses were compared for $TNF^{+/+}$ embryos with embryos homo- or heterozygous for TNF-KO obtained from the crosses of $TNF^{-/-}$ or $TNF^{+/+}$ males with TNF-KO females, or $TNF^{-/-}$ males with $TNF^{+/+}$ females. One-tailed p values were calculated with Fisher's exact test on contingency tables. Note that pre-implantation losses were also significantly lower for $TNF^{+/+}$ embryos with two-tailed Fisher's exact test ($p = 0.024$).

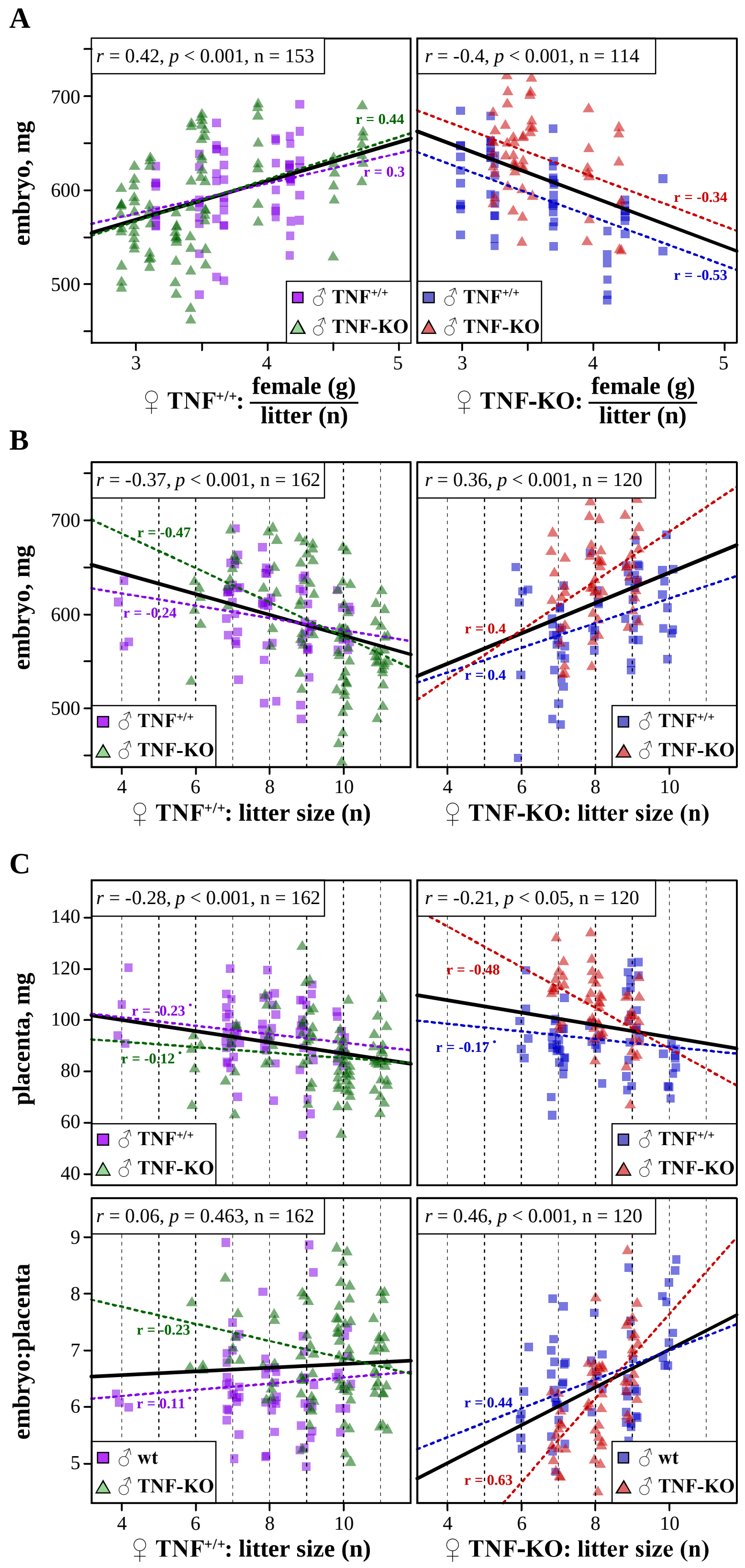

Fig. S2. Maternal TNFα is responsible for the offspring number-size trade-off.

A-B) Correlations of embryo weights with **(A)** mother weight to litter size ratio and **(B)** the number of offspring for $TNF^{+/+}$ (left panel) and $TNF^{-/-}$ (right panel) females. Regression lines are shown for matings of control females with $TNF^{+/+}$ (purple) and TNF-KO (green) males, and of TNF-KO females with control (blue) and TNF-KO (red) males. Correlation coefficients (r) for each group are also indicated in the same colour. Correlation coefficients (r), significance (p) and the number of studied embryos (n) for $TNF^{+/+}$ (left) and $TNF^{-/-}$ (right) females are shown in the top left corners and the corresponding regression lines are plotted in black. Note, that in **(A)** 5% outliers with respect to mother weight to litter size ratio were removed using minimum covariance determinant algorithm from the MASS package of R. See also Table S2 for the statistical details.

C) Correlations of placental weights (top panels) and *E*:*P* ratios (bottom panels) with the number of offspring for $TNF^{+/+}$ (left panel) and $TNF^{-/-}$ (right panel) females. Insignificant correlations are marked with dot.

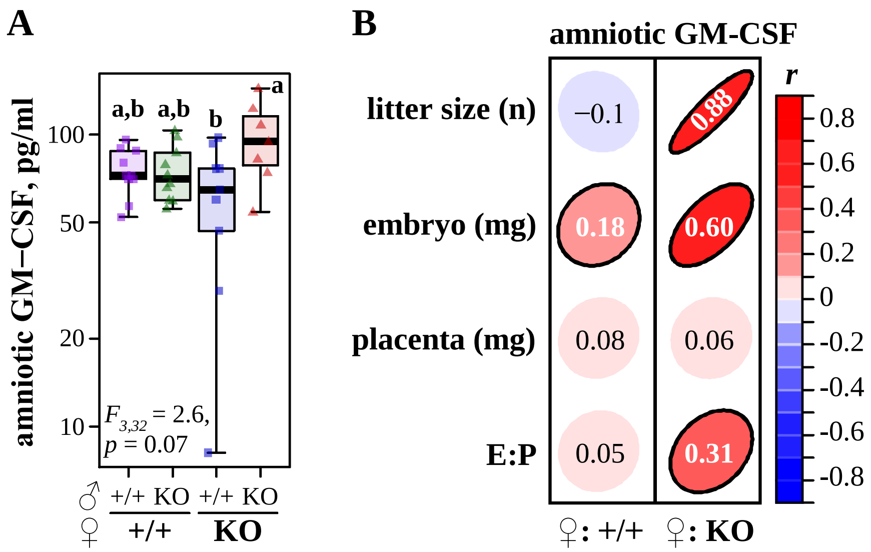

Fig. S3. TNFα modulates the levels of GM-CSF in amniotic fluid with respect to the litter size.

A) Knockout of TNFα only modestly affected the levels of GM-CSF cytokine in amniotic fluid as suggested by one-way ANOVA of $\log_{10}$-scaled concentrations. However, GM-CSF differed significantly between the matings of $TNF^{+/+}$ and $TNF^{-/-}$ males with TNF-KO females. Letters indicate statistically significant differences ($p < 0.05$) based on Least Significant Difference test (LSD). Individual and boxplot-summarized values of $\log_{10}$ GM-CSF cytokine levels are shown for females from the 4 mating groups. Boxes correspond to a range of Q1 (25%) to Q3 (75%), lines – Q2 (medians), and whickers extend to 1.5 interquartile range (IQR = Q3-Q1).

B) Heatmap of correlations of $\log_{10}$-scaled concentrations of GM-CSF with litter size, embryo and placental weights and *E*:*P* ratios for $TNF^{+/+}$ (left) and TNF-KO (right) females. See also Table S3 for statistical details. Significant correlations ($p < 0.05$) are outlined. Negative correlations are shown in blue, positive – in red.

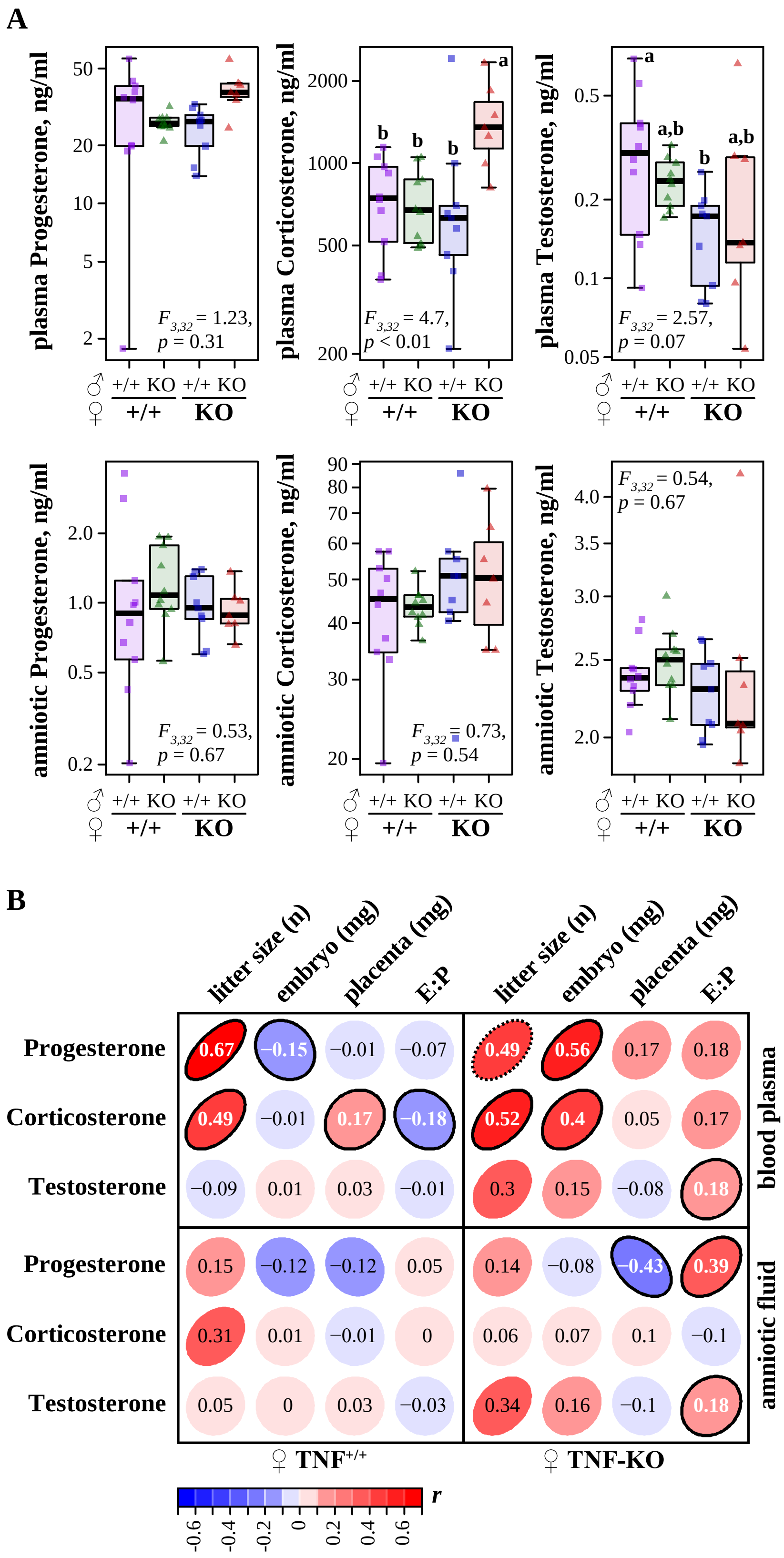

Fig. S4. TNFα is required for hormonal homeostasis of pregnancy.

A) Boxplots of log_{10}-scaled concentrations of steroid hormones (progesterone, corticosterone, testosterone) in blood plasma (top panel) and amniotic fluid (bottom panel). One-way ANOVA followed by LSD revealed a significant increase in serum corticosterone levels in TNF-KO females mated with TNF-KO males. Serum testosterone levels were marginally affected ($p < 0.1$), while the levels of other hormones remained unchanged. Letters indicate statistically significant differences ($p < 0.05$) for plasma corticosterone and testosterone.

B) Heatmap of raw correlations of steroid hormones (progesterone, corticosterone, testosterone) in serum (top panel) and amniotic fluid (bottom panel) with litter size, embryo and placental weights and *E*:*P* ratios for $TNF^{+/+}$ (left panel) and TNF-KO (right panel) females. Significant correlations ($p < 0.05$) are outlined. Dashed lines indicate marginally significant correlations ($p < 0.1$). See also Tables S4, S5 for statistical details.

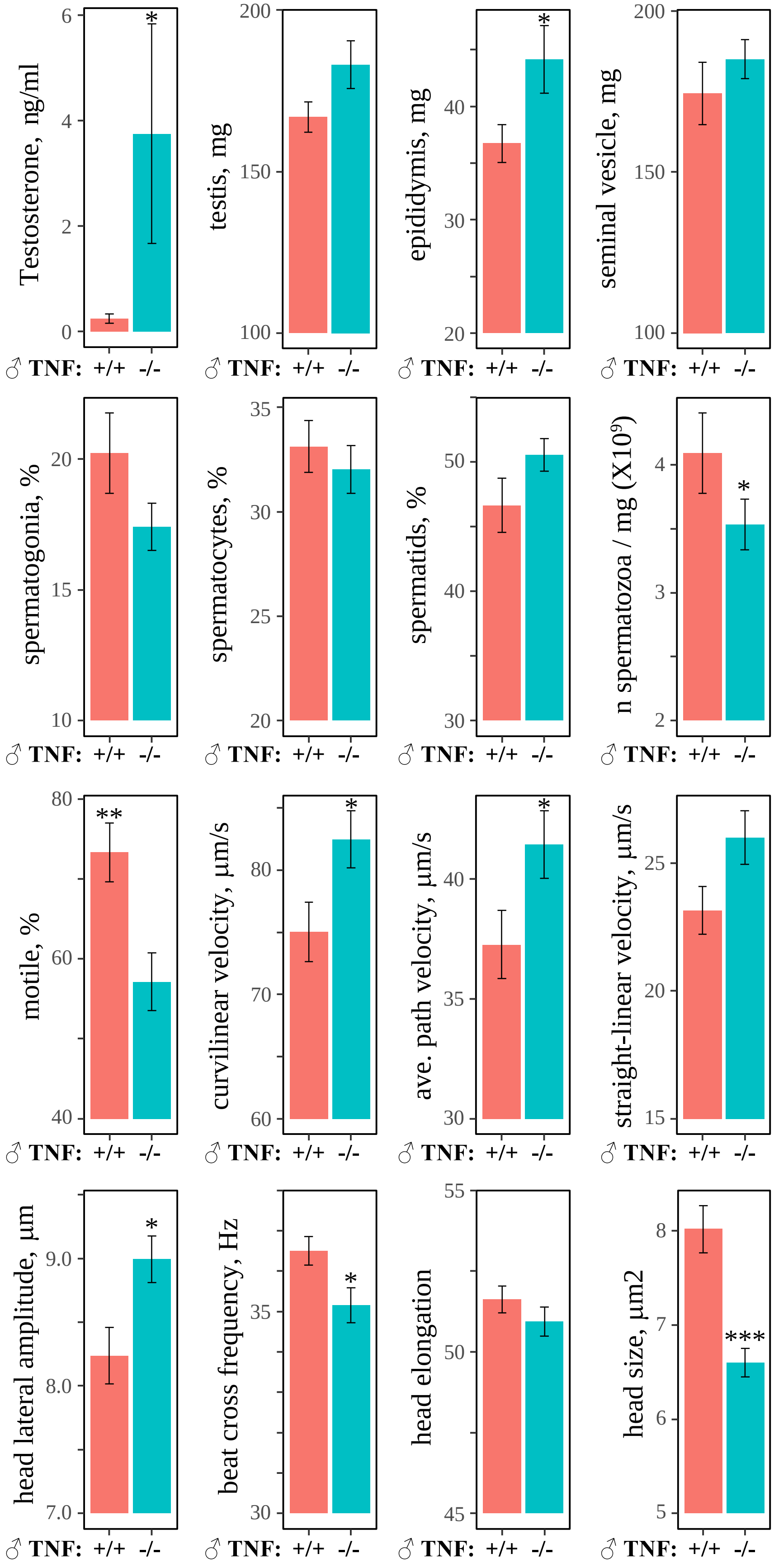
Testosterone, ng/ml
0
2
4
6
*
testis, mg
100
150
200
epididymis, mg
20
30
40
*
seminal vesicle, mg
100
150
200
spermatogonia, %
10
15
20
spermatocytes, %
20
25
30
35
spermatids, %
30
40
50
n spermatozoa / mg (X10⁹)
2
3
4
*
motile, %
40
60
80
**
curvilinear velocity, µm/s
60
70
80
*
ave. path velocity, µm/s
30
35
40
*
straight-linear velocity, µm/s
15
20
25
head lateral amplitude, µm
7.0
8.0
9.0
*
beat cross frequency, Hz
30
35
*
head elongation
45
50
55
head size, µm2
5
6
7
8
***
♂ TNF: +/+ -/-

Fig. S5. Function of TNFα in male's reproduction

Serum testosterone levels, weights of androgen-dependent tissues (testis, epididymis, seminal vesicles), sperm differentiation (% spermatogonia, spermatocytes, spermatids), the number (per mg of epididymis) of spermatozoa and percentage of motile spermatozoa, and their velocities and shape (sperm head elongation, size) characteristics were assessed for the control and TNF-KO males housed with females. All parameters except for testosterone were compared with Student's t-test, * - $p < 0.05$, ** - $p < 0.01$. Given a significant departure of log_{10}-scaled testosterone levels from the normal distribution for TNF-KO males due to a substantial increase in variations, testosterone concentrations were compared with non-parametric Mann-Whitney U-test.

Table S1. Summary of the statistical analysis of maternal and paternal effects of TNF-KO on fertility, embryo growth and reproductive output in mice.

Parameter:	Factor:		
	♀ genotype	♂ genotype	♀ genotype x ♂ genotype
Fertility:			
• ovulated eggs (n)	$F_{1,51} = 6.63$, $p < 0.05$		
• implanted embryos (n)	$F_{1,49} = 2.57$, $p = 0.11$	$F_{1,49} = 0.11$, $p = 0.74$	$F_{1,49} = 0.59$, $p = 0.45$
• live embryos (n)	$F_{1,49} = 2.53$, $p = 0.12$	$F_{1,49} = 0.03$, $p = 0.87$	$F_{1,49} = 0.45$, $p = 0.51$
Reproductive output:			
• litter size (n)	$F_{1,32} = 0.53$, $p = 0.47$	$F_{1,32} = 4.77$, $p < 0.05$	$F_{1,32} = 0.64$, $p = 0.43$
• litter weight (g)	$F_{1,32} = 0.11$, $p = 0.74$	$F_{1,32} = 6.13$, $p < 0.05$	$F_{1,32} = 0.06$, $p = 0.8$
Embryo growth:			
embryo weight (mg)	$F_{1,278} = 12.05$, $p < 0.001$	$F_{1,278} = 5.59$, $p < 0.05$	$F_{1,278} = 20.33$, $p < 0.001$
placental weight (mg)	$F_{1,278} = 27.37$, $p < 0.001$	$F_{1,278} \sim 0$, $p = 0.94$	$F_{1,278} = 42.0$, $p < 0.001$
embryo:placenta ratio	$F_{1,278} = 9.54$, $p < 0.01$	$F_{1,278} = 3.38$, $p = 0.07$	$F_{1,278} = 13.78$, $p < 0.001$

The number of ovulated eggs was estimated from the number of corpora lutea (CL) and one-way ANOVA showed a significant effect of TNF-KO on ovulation. Main effects and interaction of the parental genotypes ($TNF^{+/+}$ or $TNF^{-/-}$) on the number of implanted embryos (sum of live and resorbed embryos), live embryos, reproductive output (litter size, weight) and embryo growth were assessed by two-way ANOVA (F-statistics and p values are shown). Reproductive output was calculated only for females carrying live embryos at day 16.5 of gestation.

Table S2. Maternal and paternal effects of TNF-KO along with covariates (mother weight to litter size ratio or the number of offspring) on embryo and placental weight, and embryo to placenta weight ratio in mice.

Parameter:	Covariate (Z):	Factor:				
		♀ x ♂	*Z*	♀ x *Z*	♂ x Z	♀ x ♂ x *Z*
embryo, mg	M_{female}:L	$F_{3,259}$=26.31, p < 0.001	$F_{1,259}$=2.86, p = 0.09	$F_{1,259}$=40.5, p < 0.001	$F_{1,259}$=0.32, p = 0.57	$F_{1,259}$=0.24, p = 0.63
embryo, mg	*L*	$F_{3,274}$=19.59, p < 0.001	$F_{1,274}$=3.34, p = 0.07	$F_{1,274}$=32.8, p < 0.001	$F_{1,274}$=2.48, p = 0.12	$F_{1,274}$=5.62, p < 0.05
E:*P* ratio	*L*	$F_{3,274}$=13.76, p < 0.001	$F_{1,274}$=4.56, p < 0.05	$F_{1,274}$=20.94, p < 0.001	$F_{1,274}$=0.71, p = 0.4	$F_{1,274}$=13.68, p < 0.001
placenta, mg	*L*	$F_{3,274}$=11.8, p < 0.001	$F_{1,274}$=11.21, p < 0.01	$F_{1,274}$=1.61, p = 0.2	$F_{1,274}$=0.58, p = 0.45	$F_{1,274}$=6.36, p < 0.05

Variations in embryo, placental weights (mg) and embryo to placenta weight ratios (*E*:*P*) with respect to parental genotype and linear covariates (*Z*) were modelled as: *Y* ~ ♀ x ♂ + *Z* + ♀ x *Z* + ♂ x *Z* + ♀ x ♂ x *Z*, where ♀, ♂ - parental genotypes ($TNF^{+/+}$ or $TNF^{-/-}$), *Z* - mother weight to litter size ratio (M_{female}:*L*) or the litter size (*L*). ANCOVA revealed 1) significant effects of parental genotype on all response variables and 2) interaction effects of ♀ x *Z* on embryo weight and *E*:*P* ratio. The latter is due to a switch in correlation signs or gain in correlation between embryo weight or *E*:*P* ratio and the covariates in TNF-KO females as compared to $TNF^{+/+}$ females (Figure 1, S1).

Table S3. Maternal and paternal effects of TNF-KO along with covariates (litter size, embryo weight, placental weight or *E*:*P* ratio) on the $\log_{10}$ levels (g/l) of GM-CSF in amniotic fluid.

Parameter:	Covariate (Z):	Factor:				
		♀ x ♂	*Z*	♀ x *Z*	♂ x Z	♀ x ♂ x *Z*
GM-CSF	*litter size (n)*	$F_{3,28}$=19.21, $p < 0.001$	$F_{1,28}$=26.03, $p < 0.001$	$F_{1,28}$=35.71, $p < 0.001$	$F_{1,28}$=0.13, $p = 0.72$	$F_{1,28}$=0.15, $p = 0.7$
	embryo, mg	$F_{3,274}$=15.63, $p < 0.001$	$F_{1,274}$=41.72, $p < 0.001$	$F_{1,274}$=22.41, $p < 0.001$	$F_{1,274}$=1.1, $p = 0.3$	$F_{1,274}$=10.92, $p < 0.01$
	E:P ratio	$F_{3,274}$=22.11, $p < 0.001$	$F_{1,274}$=20.9, $p < 0.001$	$F_{1,274}$=20.38, $p < 0.001$	$F_{1,274}$ ~ 0, $p = 0.95$	$F_{1,274}$=0.57, $p = 0.45$
	placenta, mg	$F_{3,274}$=13.17, $p < 0.001$	$F_{1,274}$=0.9, $p = 0.34$	$F_{1,274}$=5.48, $p < 0.05$	$F_{1,274}$=1.65, $p = 0.2$	$F_{1,274}$=11.18, $p < 0.001$

Variations in amniotic $\log_{10}$ GM-CSF levels with respect to parental genotype and linear covariates (*Z*) were modelled as: *Y* ~ ♀ x ♂ + *Z* + ♀ x *Z* + ♂ x *Z* + ♀ x ♂ x *Z*, where ♀, ♂ - parental genotypes ($TNF^{+/+}$ or $TNF^{-/-}$), *Z* – litter size (n), embryo or placental weight (mg), or *E*:*P* weight ratio. ANCOVA revealed significant interaction effects of maternal genotype and covariates: litter size, embryo weight and *E*:*P* on GM-CSF levels in amniotic fluid (Figure 2, S2). Effect of parental genotype (♀ x ♂) on GM-CSF was insignificant in the absence of covariates (Figure S2).

Table S4. Maternal and paternal effects of TNF-KO along with covariates on the log_{10} levels (g/l) of blood plasma progesterone, corticosterone and testosterone.

Parameter:	Covariate (Z):	Factor:				
		♀ x ♂	*Z*	♀ x *Z*	♂ x Z	♀ x ♂ x *Z*
Progesterone	*litter size (n)*	$F_{3,28}$=10.71, $p < 0.001$	$F_{1,28}$=33.13, $p < 0.001$	$F_{1,28}$=16.52, $p < 0.001$	$F_{1,28}$=14.77, $p < 0.001$	$F_{1,28}$=1.65, $p = 0.21$
	embryo, mg	$F_{3,274}$=22.03, $p < 0.001$	$F_{1,274}$ ~ 0, $p = 0.94$	$F_{1,274}$=20.71, $p < 0.001$	$F_{1,274}$=2.04, $p = 0.15$	$F_{1,274}$=6.67, $p < 0.05$
	E:P ratio	$F_{3,274}$=18.45, $p < 0.001$	$F_{1,274}$=2.68, $p = 0.1$	$F_{1,274}$=5.37, $p < 0.05$	$F_{1,274}$=0.02, $p = 0.89$	$F_{1,274}$=0.04, $p = 0.84$
	placenta, mg	$F_{3,274}$=15.83, $p < 0.001$	$F_{1,274}$=2.44, $p = 0.12$	$F_{1,274}$ ~ 0, $p = 0.95$	$F_{1,274}$ ~ 0, $p = 0.95$	$F_{1,28}$=2.0, $p = 0.12$
Corticosterone	*litter size (n)*	$F_{3,28}$=3.44, $p < 0.05$	$F_{1,28}$=10.58, $p < 0.01$	$F_{1,28}$=0.22, $p = 0.64$	$F_{1,28}$=0.02, $p = 0.88$	$F_{1,28}$=0.8, $p = 0.38$
	embryo, mg	$F_{3,274}$=23.02, $p < 0.001$	$F_{1,274}$=2.02, $p = 0.16$	$F_{1,274}$=7.15, $p < 0.01$	$F_{1,274}$=7.65, $p < 0.01$	$F_{1,274}$=1.86, $p = 0.17$
	E:P ratio	$F_{3,274}$=31.94, $p < 0.001$	$F_{1,274}$=2.56, $p = 0.11$	$F_{1,274}$=20.16, $p < 0.001$	$F_{1,274}$=0.02, $p = 0.88$	$F_{1,274}$=4.67, $p < 0.05$
	placenta, mg	$F_{3,274}$=29.25, $p < 0.001$	$F_{1,274}$=1.62, $p = 0.2$	$F_{1,274}$=12.22, $p < 0.001$	$F_{1,274}$=1.12, $p = 0.29$	$F_{1,274}$=2.82, $p = 0.09$
Testosterone	*litter size (n)*	$F_{3,28}$=1.21, $p = 0.32$	$F_{1,28}$=0.55, $p = 0.46$	$F_{1,28}$=0.79, $p = 0.38$	$F_{1,28}$=0.05, $p = 0.83$	$F_{1,28}$=0.15, $p = 0.7$
	embryo, mg	$F_{3,274}$=3.51, $p < 0.05$	$F_{1,274}$=0.94, $p = 0.33$	$F_{1,274}$=2.28, $p = 0.13$	$F_{1,274}$=1.96, $p = 0.16$	$F_{1,274}$=2.01, $p = 0.16$
	E:P ratio	$F_{3,274}$=5.5, $p < 0.01$	$F_{1,274}$=4.26, $p < 0.05$	$F_{1,274}$=3.66, $p = 0.06$	$F_{1,274}$=0.67, $p = 0.41$	$F_{1,274}$=0.41, $p = 0.52$
	placenta, mg	$F_{3,274}$=2.83, $p < 0.05$	$F_{1,274}$=2.16, $p = 0.14$	$F_{1,274}$=1.54, $p = 0.21$	$F_{1,274}$=0.11, $p = 0.74$	$F_{1,274}$ ~ 0, $p = 0.97$

Table S5. Maternal and paternal effects of TNF-KO along with covariates on the log_{10} levels (g/l) of progesterone, corticosterone and testosterone in amniotic fluid.

Parameter:	Covariate (Z):	Factor:				
		♀ x ♂	*Z*	♀ x *Z*	♂ x Z	♀ x ♂ x *Z*
Progesterone	*litter size (n)*	$F_{3,28}$=0.18, p = 0.91	$F_{1,28}$=0.19, p = 0.67	$F_{1,28}$ ~ 0, p = 0.98	$F_{1,28}$=0.02, p = 0.89	$F_{1,28}$=0.07, p = 0.79
	embryo, mg	$F_{3,274}$=2.18, p = 0.09	$F_{1,274}$ = 2.1, p = 0.15	$F_{1,274}$=0.39, p = 0.53	$F_{1,274}$=0.13, p = 0.71	$F_{1,274}$=1.4, p = 0.24
	E:P ratio	$F_{3,274}$=4.0, p < 0.01	$F_{1,274}$=2.46, p = 0.12	$F_{1,274}$=5.54, p < 0.05	$F_{1,274}$=0.38, p = 0.54	$F_{1,274}$=6.38, p < 0.05
	placenta, mg	$F_{3,274}$=2.9, p < 0.05	$F_{1,274}$=6.7, p < 0.05	$F_{1,274}$=4.33, p < 0.05	$F_{1,274}$=1.57, p = 0.21	$F_{1,274}$=9.97, p < 0.01
Corticosterone	*litter size (n)*	$F_{3,28}$=0.3, p = 0.82	$F_{1,28}$=0.92, p = 0.35	$F_{1,28}$=0.21, p = 0.65	$F_{1,28}$=0.08, p = 0.78	$F_{1,28}$=3.62, p < 0.07
	embryo, mg	$F_{3,27}$=1.02, p = 0.38	$F_{1,27}$=0.09, p = 0.76	$F_{1,27}$=0.22, p = 0.64	$F_{1,27}$=2.6, p = 0.11	$F_{1,27}$=1.51, p = 0.22
	E:P ratio	$F_{3,274}$=3.49, p < 0.05	$F_{1,274}$=0.76, p = 0.38	$F_{1,274}$=0.34, p = 0.56	$F_{1,274}$=8.9, p < 0.01	$F_{1,274}$=17.65, p < 0.001
	placenta, mg	$F_{3,274}$=1.57, p = 0.19	$F_{1,274}$=0.3, p = 0.58	$F_{1,274}$=0.09, p = 0.76	$F_{1,274}$=3.62, p = 0.06	$F_{1,274}$=10.66, p < 0.001
Testosterone	*litter size (n)*	$F_{3,28}$=0.74, p = 0.54	$F_{1,28}$=0.89, p = 0.35	$F_{1,28}$=1.72, p = 0.2	$F_{1,28}$=0.07, p = 0.79	$F_{1,28}$=0.03, p = 0.87
	embryo, mg	$F_{3,274}$=2.07, p = 0.1	$F_{1,274}$=2.69, p = 0.1	$F_{1,274}$=2.12, p = 0.15	$F_{1,274}$=0.77, p = 0.38	$F_{1,274}$=2.26, p = 0.13
	E:P ratio	$F_{3,274}$=3.06, p < 0.05	$F_{1,274}$=1.42, p = 0.23	$F_{1,274}$=7.67, p < 0.01	$F_{1,274}$=0.5, p = 0.48	$F_{1,274}$=4.84, p < 0.05
	placenta, mg	$F_{3,274}$=3.06, p < 0.05	$F_{1,274}$=0.34, p = 0.56	$F_{1,274}$=5.65, p < 0.05	$F_{1,274}$=4.51, p < 0.05	$F_{1,274}$=15.31, p < 0.001
